# Molecular implications of *HOX* genes targeting multiple signaling pathways in cancer

**DOI:** 10.1007/s10565-021-09657-2

**Published:** 2021-10-06

**Authors:** U Sangeetha Shenoy, Divya Adiga, Shama Prasada Kabekkodu, Keith D Hunter, Raghu Radhakrishnan

**Affiliations:** 1 Department of Cell and Molecular Biology, Manipal School of Life Sciences, Manipal Academy of Higher Education, Manipal, Karnataka 576104 India; 2 Academic Unit of Oral and Maxillofacial Medicine and Pathology, School of Clinical Dentistry, University of Sheffield, Sheffield, S10 2TA UK; 3 Department of Oral Pathology, Manipal College of Dental Sciences, Manipal, Manipal Academy of Higher Education, Manipal, 576104 India

**Keywords:** *HOX* genes, Diagnostic and prognostic significance, Signaling pathways, Metastasis, Cancer

## Abstract

Homeobox (*HOX*) genes encode highly conserved homeotic transcription factors that play a crucial role in organogenesis and tissue homeostasis. Their deregulation impacts the function of several regulatory molecules contributing to tumor initiation and progression. A functional bridge exists between altered gene expression of individual *HOX* genes and tumorigenesis. This review focuses on how deregulation in the HOX-associated signaling pathways contributes to the metastatic progression in cancer. We discuss their functional significance, clinical implications and ascertain their role as a diagnostic and prognostic biomarker in the various cancer types. Besides, the mechanism of understanding the theoretical underpinning that affects *HOX*-mediated therapy resistance in cancers has been outlined. The knowledge gained shall pave the way for newer insights into the treatment of cancer.

**Graphical abstract:**

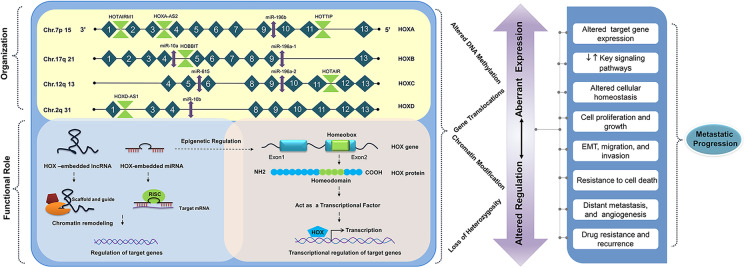

## Introduction

Distinct classes of regulatory molecules govern the functional activities in a normal cell through the interaction with different signaling pathways. Any disruption of intercellular and intracellular signaling networks has wider implications on the cellular function resulting in many diseases including cancer (Derakhshani et al. 2020; Sever and Brugge 2015). Accumulating evidence demonstrates that homeobox (*HOX*) genes that function as master regulators during embryogenesis have roles in both organogenesis and oncogenesis (Smith et al. 2019). The vertebrate HOX clusters contain the protein-coding *HOX* genes as well as non-coding RNAs (ncRNAs) (Botti et al. 2018). Studies have demonstrated that the expression of the *HOX* genes is deregulated in cancer either due to temporospatial heterogeneity, gene dominance, genetic, or epigenetic mechanisms (Haria and Naora 2013; Li et al. 2019a). The aberrant expression of the *HOX* gene loci disrupts the dynamic process of normal intracellular signaling, thereby triggering cellular dysfunction and impaired tumor immune surveillance. The prolonged activation of crucial signaling pathways controlling cell growth, cell cycle, and cell proliferation creates a favorable microenvironment for tumor growth (Giancotti 2014). This review attempts to summarize the existing knowledge of deregulated *HOX* genes and their role in modulating the key signaling pathways of cancer with a special focus on metastatic progression. The molecular basis by which the tumor cells acquire different cancer hallmarks and the role of *HOX* genes as a prognostic marker and therapeutic target is widely explored.

## HOX cluster: origin, evolution, and organization

The *HOX* genes were first discovered in *Drosophila melanogaster*, due to two mutations mapped to the Antennapedia and Bithorax complex on separate chromosomes. These mutations are often referred to as homeotic transformations, specifying the change of segment identity along the anteroposterior axis. Initially, it was believed that a single proto-*HOX* gene of primitive organisms had undergone duplication and divergence to form a gene cluster in multicellular organisms (Ferrier and Holland 2001; Holland 2013). However, based on the two rounds (2R) of the whole-genome duplication (WGD) hypothesis, one-to-four transition in the human HOX cluster occurred by three-step sequential events of segmental duplications, independent gene duplications, and translocations (Abbasi 2015). Humans have 39 *HOX* genes, which are arranged in four clusters, namely, HOXA, HOXB, HOXC, and HOXD (Mallo 2018), mapped onto the specific chromosome loci (7p15, 17q21, 12q13, and 2q31). Each cluster has 13 paralog groups, each having an equivalent position in the cluster, numbered from 1 to 13 in the 3′ to 5′ direction (Apiou et al. 1996).

*HOX* genes are small, having two exons and a single intron. The second exon contains a homeobox region that has a high degree of homology within the paralog groups. It gets translated into a highly conserved helix-turn-helix DNA binding protein termed as “homeodomain” of 60 amino acid length (Lappin et al. 2006). Incidentally, the conservation of HOX clusters among different species is unique. Phylogenetic analysis reveals that the putative regulatory elements in the HOX cluster are evolutionarily conserved among those different species, which are separated by approximately 500 million years of evolution (Santini et al. 2003). Further, it was found that homology in non-coding sequences in different vertebrate species paved the way to explore the regulatory regions other than the protein-coding genes in the HOX cluster (Matsunami et al. 2010). Embedded within the HOX cluster are several ncRNAs. Rinn et al. described 231 ncRNAs inside the HOX cluster network (Rinn et al. 2007), of which 15% have been functionally characterized.

## *HOX* genes and human health

Since their discovery, the regulation of the *HOX* genes and their function has been extensively studied. The *HOX* genes are first expressed in the developing embryo at the gastrulation stage (Boncinelli and Mallamaci 1995). *HOX* genes that are located near the 3′ of the cluster are expressed earlier than those that are located near the 5′, suggesting that the activation of *HOX* genes is collinear with its position, a property referred to as the principle of collinearity (Gaunt 2018). A group of cells called the functional domain requires positional information for their differentiation, which means that the *HOX* genes that are expressed in functional domains are committed to form specific body parts (Kmita and Duboule 2003). Besides, *HOX* genes have a crucial role in the formation of skeletal morphology (Song et al. 2020), limb (Pineault and Wellik 2014), and organ development (Hombría and Lovegrove 2003).

*HOX* genes act as transcription factors that usually complex with co-factors to activate genes involved in cell proliferation, adhesion, differentiation, cell migration, and cell death under normal physiological conditions through their homeobox domain (Hombría and Lovegrove 2003; Moens and Selleri 2006). Thus, the impact of *HOX* gene deregulation at various stages of development leads to abnormalities in skeletal morphology such as hand-foot-genital syndrome, syndactyly, Guttmacher syndrome, congenital heart diseases, retinal degenerative diseases, and cancer (Lescroart and Zaffran 2018; Quinonez and Innis 2014; Shah and Sukumar 2010; Zagozewski et al. 2014). This review focuses on the role of *HOX* gene deregulation in cancer.

## Regulation of *HOX* genes in cancer

**Fig. 1:**
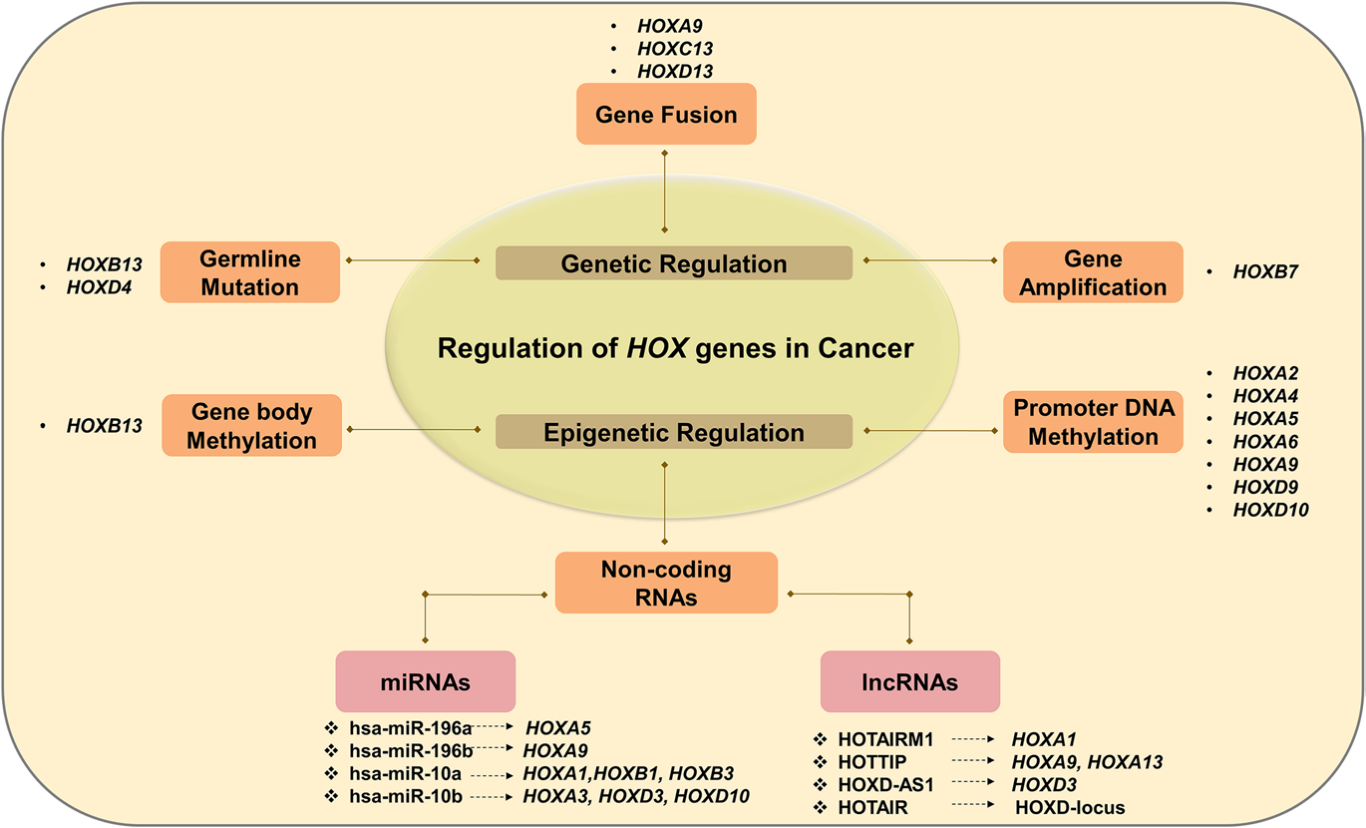
*HOX* gene regulation in cancer modulated by genetic and epigenetic mechanisms

Several researchers have reviewed the role of *HOX* genes in the normal and tumor microenvironment (Bhatlekar et al. 2018). Meanwhile, *HOX* gene regulation is tissue-specific, and their deregulation varies from one cancer to another. They either act as oncogenes or tumor suppressors (Table 1) in a context-dependent manner. Functional studies have revealed that both genetic and epigenetic factors contribute to the abnormal expression of *HOX* genes in different cancers (Li et al. 2019a). The different modes of regulation of *HOX* genes in cancer have been outlined in Fig. 1.

**Table 1:** The role of *HOX* genes in the development of human cancer

Cluster	*HOX* genes	Chromosomal location	Cancer	Aberrant expression	References
Oncogenic *HOX* genes					
HOXA cluster	*HOXA1*	chr7:27,092,993–27,096,000	Gastric cancer, human mammary carcinoma, prostate cancer	**↑**	Hou et al. 2012; Mohankumar et al. 2007; Wang et al. 2015; Yuan et al. 2016
	*HOXA10*	chr7:27,170,605–27,174,320	Bladder cancer, colorectal cancer, acute myeloid leukemia, laryngeal squamous cell cancer, pancreatic cancer	**↑**	Cui et al. 2014; Guo et al. 2020; Li et al. 2020a; Liu et al. 2019a; Yuan et al. 2018
	*HOXA13*	chr7:27,194,364–27,200,091	Gastric cancer, lung squamous cancer, bladder cancer, kidney renal clear-cell carcinoma, glioma	**↑**	Cui et al. 2020; Duan et al. 2015; Han et al. 2018; Hu et al. 2017; Zhang et al. 2017
HOXB cluster	*HOXB2*	chr17:48,542,655–48,544,989	Glioblastoma	**↑**	Li et al. 2020b
	*HOXB3*	chr17:48,550,006–48,590,272	High-grade serous ovarian carcinoma	**↑**	Miller et al. 2018
	*HOXB4*	chr17:48,575,507–48,578,350	Chronic myelogenous leukemia	**↑**	Wang et al. 2016
	*HOXB5*	chr17:48,591,257–48,593,961	Breast cancer, gastric cancer, non-small cell lung cancer, head and neck squamous cell carcinoma, pancreatic cancer, retinoblastoma, hepatocellular carcinoma	**↑**	Gao et al. 2020; Hong et al. 2015; Lee et al. 2015; Lee et al. 2020; Su et al. 2020; Xu et al. 2018; Zhang et al. 2018
	*HOXB7*	chr17:48,607,232–48,611,017	Esophageal squamous cell carcinoma, gastric cancer, glioma, cutaneous squamous cell carcinoma, breast cancer, pancreatic cancer, hepatocellular carcinoma, acute lymphoblastic leukemia, multiple myeloma, lung adenocarcinoma, colorectal cancer	**↑**	Gao and Chen 2018; He et al. 2017; Huo et al. 2019; Li et al. 2015; Liao et al. 2011; Liu et al. 2015a; Storti et al. 2011; Tsuboi et al. 2017; Wang et al. 2017; Zhong et al. 2019; Zhuang et al. 2015
	*HOXB8*	chr17:48,612,346–48,615,292	Colorectal cancer, osteosarcoma	**↑**	Guo et al. 2019; Wang et al. 2019a
HOXC cluster	*HOXC6*	chr12:54,028,440–54,030,823	Gastric cancer, nasopharyngeal carcinoma, cervical cancer, glioblastoma, head and neck squamous cell carcinoma	**↑**	Chang et al. 2017; Jung et al. 2020; Moon et al. 2012; Wang et al. 2019b; Yang et al. 2019
	*HOXC8*	chr12:54,008,985–54,012,769	Laryngeal squamous cell carcinoma, glioma, cervical cancer, non-small cell lung cancer	**↑**	de Barros E Lima Bueno et al. 2016; Huang et al. 2018; Liang et al. 2018; Liu et al. 2018
	*HOXC9*	chr12:53,995,509–54,003,298	Colorectal cancer, gastric cancer, neuroblastoma, glioblastoma	**↑**	Hu et al. 2019; Kocak et al. 2013; Xuan et al. 2016; Zhao et al. 2020
	*HOXC10*	chr12:53,985,146–53,990,279	Thyroid cancer, gastric cancer, glioma, breast cancer	**↑**	Feng et al. 2015; Kim et al. 2019; Li et al. 2018; Miwa et al. 2019; Sadik et al. 2016
	*HOXC11*	chr12:53,973,126–53,977,643	Renal cell carcinoma	**↑**	Liu et al. 2015b
HOXD cluster	*HOXD3*	chr2:176,160,891–176,173,085	Non-small cell lung cancer	**↑**	Miyazaki et al. 2002
	*HOXD4*	chr2:176,151,550–176,153,226	Gastric adenocarcinoma	**↑**	Liu et al. 2019b
	*HOXD9*	chr2:176,122,719–176,124,937	Gastric cancer	**↑**	Xiong et al. 2020
	*HOXD11*	chr2:176,104,216–176,109,754	Laryngeal squamous cell carcinoma	**↑**	de Barros E Lima Bueno et al. 2016
Tumor suppressor *HOX* genes					
HOXA cluster	*HOXA3*	chr7:27,107,012–27,140,219	Lung adenocarcinoma	↓	Gan et al. 2018
	*HOXA5*	chr7:27,141,052–27,143,681	Cervical cancer, osteosarcoma, breast cancer, gastric cancer, non-small cell lung cancer, colorectal cancer	↓	Chen et al. 2004; Chen et al. 2019; Ma et al. 2020; Ordóñez-Morán et al. 2015; Peng et al. 2018; Raman et al. 2000; Wang et al. 2019c; Zhang et al. 2015
HOXD cluster	*HOXD1*	chr2:176,188,668–176,190,907	Kidney renal clear-cell carcinoma	↓	Cui et al. 2021
	*HOXD8*	chr2:176,130,357–176,132,000	Colorectal cancer	↓	Mansour and Senga 2017
	*HOXD13*	chr2:176,092,721–176,095,944	Prostate cancer	↓	Xu et al. 2021
*HOX* genes with both oncogenic and tumor suppressor activity					
HOXA cluster	*HOXA4*	chr7:27,128,507–27,130,780	High-grade serous ovarian carcinoma	**↑**	Miller et al. 2018
			Lung cancer	↓	Cheng et al. 2018
	*HOXA9*	chr7:27,162,438–27,165,537	Acute myeloid leukemia, acute lymphoblastic leukemia, ovarian cancer	**↑**	de Bock et al. 2018; Brumatti et al. 2013; Ko et al. 2012; Zhao et al. 2015
			Cervical cancer, cutaneous squamous cell carcinoma	↓	Alvarado-Ruiz et al. 2016; Han et al. 2019
	*HOXA11*	chr7:27,181,157–27,185,232	Laryngeal squamous cell cancer	**↑**	Li et al. 2020a
			Glioblastoma, breast cancer	↓	Se et al. 2017; Xia et al. 2017
HOXB cluster	*HOXB9*	chr17:48,621,156–48,626,358	Breast cancer, hepatocellular carcinoma, oral squamous cell carcinoma, laryngeal squamous cell carcinoma	**↑**	Hayashida et al. 2010; Sha et al. 2015; Sun et al. 2017; Xue et al. 2017
			Gastric cancer	↓	Zhang et al. 2019
	*HOXB13*	chr17:48,724,763–48,728,750	Urinary bladder cancer, breast cancer, prostate cancer	**↑**	Kim et al. 2014; Marra et al. 2013; Shah et al. 2013
			Colorectal cancer	↓	Jung et al. 2005
HOXD cluster	*HOXD10*	chr2:176,116,778–176,119,937	Laryngeal squamous cell carcinoma	**↑**	de Barros E Lima Bueno et al. 2016
			Colon cancer, hepatocellular carcinoma, cholangiocellular carcinoma	↓	Guo et al. 2017; Yang et al. 2015; Yuan et al. 2019

NOTE: ↑, upregulated; ↓, downregulated

### Genetic regulation of *HOX* genes in cancer

The genetic alterations at the *HOX* gene loci promote cancer progression. In this section, we outline the genetic alterations associated with *HOX* genes upon an extensive literature search.

#### ***Missense mutations***

Among the germline mutations analyzed in the HOX paralogous groups from 4 to 13, the *HOXD4* missense mutation was seen in the patients of acute lymphoblastic leukemia (ALL). The transcriptional activity of p.Glu81Val mutant *HOXD4* was observed to be 40% lower compared to the wild-type protein, leading to its partial loss of function causing ALL susceptibility. Due to the mutation, *HOXD4* lost its ability to regulate target genes involved in hematopoietic cell proliferation and differentiation (van Scherpenzeel Thim et al. 2005).

*HOXB13* is one of the widely studied genes in prostate cancer (PCa) progression (Kim et al. 2014; Yao et al. 2019). Inherited mutations in the *HOXB13* gene contribute significantly to the increased risk of PCa. Screening of more than 200 germline genes at 17q21-22 loci, in 94 PCa patients, recognizes this region as cancer-susceptible loci. The occurrence of rare and recurrent mutation G84E in *HOXB13* (rs138213197), along with its variants (*Y88D*, *L144P*, *G216C*, and *R229G*), has been implicated in hereditary PCa. The mutation rate was significant in men with familial PCa (3.1%) than non-familial PCa (0.6%). Overall, men with PCa carried the *HOXB13-G84E* allele (frequency 1.4%) when compared to normal patients (frequency 0.1%) (Ewing et al. 2012). The variant *HOXB13-G84E* has also shown an increased risk for leukemia and bladder cancer (Beebe-Dimmer et al. 2015). Understanding the effect of mutations in rare cases would pave the way to understand the pathways related to cancer progression. Another study examined the functional implication of *HOXB13* *rs138213197* (*G84E*) and *CIP2A* *rs2278911* (*R229Q*) germline variants in PCa. The interplay between *HOXB13* and *CIP2A* variants was most frequent in familial PCa (*p* < 0.001). HOXB13 protein promotes the transcription of *CIP2A*, by binding to its promoter region. The co-occurrence of this event is associated with enhanced cancer cell growth, proliferation, and migration. Thus, these allelic variants contribute to high cancer risk and aggressiveness (Sipeky et al. 2018).

While appraising the association between *HOXB13-pGly84Glu* mutation and colorectal cancer (CRC) patients in two different population registries, 0.48% of the cases had *HOXB13* mutation when compared to the control group (0.17%) (Akbari et al. 2013).

#### ***Gene fusion***

*HOX* genes act as a fusion partner of nucleoporin 98 kDa (NUP98) to drive cancer progression. The fusion protein bears the N-terminal of NUP98 with unique potential for transcriptional activation (Gough et al. 2011). The chromosomal translocation of *t* (7; 11) (p15; p15) results in the chimeric protein NUP98-HOXA9 (NHA9). The impact of gene fusion in driving the leukemic transformation of primary human CD34 + hematopoietic cells in acute myeloid leukemia (AML) has been studied (Takeda et al. 2006). NHA9 interacts with mixed-lineage leukemia 1 (MLL1) to promote proliferation by activating a large number of oncogenes (Takeda et al. 2006; Xu et al. 2016). Further, gene translocation at *t* (11;12) (p15;q13) generated a NUP98/HOXC13 fusion protein. The study showed that the fusion that occurred between the 16th exon of the *NUP98* gene and the 2nd exon of *HOXC13* might have pathogenic importance in AML (Panagopoulos et al. 2003). Recently, it was reported that fusion protein NUP98–HOXD13 (NHD13) promoted T cell acute lymphoblastic leukemia (T-ALL) by inducing the self-renewal of T cell progenitors in the thymus at the DN2 developmental stage of T cells (Shields et al. 2019). NHD13 was found to be regulated by T cell oncogenes *LMO2* and *LYL1* for self-renewal of thymocyte cells (Shields et al. 2019).

#### ***Amplification***

Meanwhile, reports on copy number variations including amplifications and deletions located in *HOX* locus are just emerging. In breast cancer (BC), comparative genomic hybridization (CGH) was performed to identify copy number alterations. Interestingly, a novel amplicon at *HOXB7* (17q21.3) was observed in 10.2% of patients whose expression was significantly correlated with poor prognosis (Hyman et al. 2002).

**Fig. 2:**
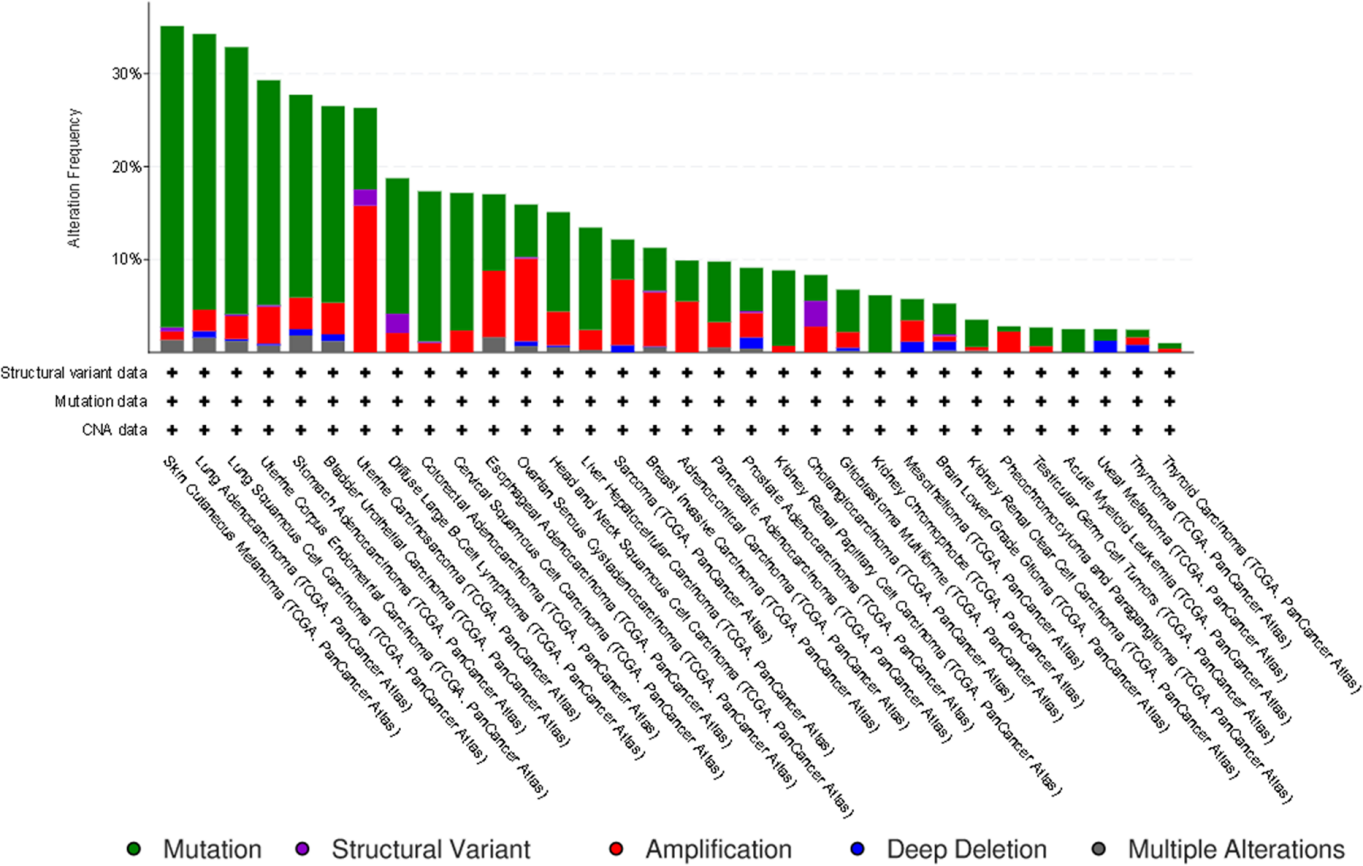
Genetic alterations of *HOX* genes: the in silico pan-cancer analysis of all the 39 *HOX* genes, across 32 TCGA cancer types performed using cBioportal. The green bars indicate mutation, violet bars indicate structural variant, red bars indicate amplification, blue bars indicate deep deletion, and gray bars indicate multiple alterations

Further, the in silico pan-cancer analysis of all the 39 *HOX* genes, performed using cBioportal (https://www.cbioportal.org/) (Cerami et al. 2012; Gao et al. 2013), showed different kinds of genetic changes such as mutations, structural variants, amplification, and deep deletions across 32 TCGA cancer types analyzed in 10,967 tissue samples (Fig. 2).

### Epigenetic regulation of *HOX* genes in cancer

Epigenetic factors associated with cancer progression include aberrant methylation at CpG sites, altered chromatin modifications, and aberrant expression of ncRNAs at HOX cluster network (Li et al. 2019a). The characterization of the epigenome thus helps to predict the biological behavior of the tumor, predict disease outcome, and plan suitable treatment regimens.

#### ***Promoter DNA methylation***

Aberrant gene methylation is the most common epigenetic alteration that not only promotes cancer progression but also confers resistance to anticancer therapies (Romero-Garcia et al. 2020). The promoter DNA methylation could prevent the binding of RNA polymerases and other transcription factors to suppress the transcriptional activation (Curradi et al. 2002). However, this gene silencing mechanism is context-dependent (Smith et al. 2020). Paço et al. (2020) have extensively reviewed the impact of promoter DNA methylation on the expression of *HOX* genes in different cancers (Paço et al. 2020b). Most recent studies that emphasized the impact of *HOX* gene promoter hypermethylation in different cancers have been briefly summarized in Table 2. In contrast, a study on BC has delineated the fact that *HOXA9-HOXA10* methylation and their promoter–promoter interaction are responsible for cancer progression. Interestingly, the CpG islands of the *HOXA10* gene functioned as an enhancer for the promoter region of the *HOXA9* gene to promote its gene expression (Park et al. 2017). In addition to the promoter DNA methylation, the altered gene expression is also attributable to the gene body methylation. Su et al. in 2018 elucidated a novel mechanism of gene body hypermethylation and homeobox oncogene activation through performing the pan-cancer analysis of 4174 genome-wide datasets and whole-genome bisulfite sequencing in 30 normal tissues and 35 tumor tissues (Su et al. 2018).

**Table 2:** Epigenetic regulation of *HOX* genes in cancer

*Promoter DNA hyper methylation*					
Cancer	*HOX* genes	Frequency	Specificity	Role	References
Breast cancer	*HOXA4*	46.6%	-	Biomarker for early cancer detection	Li et al. 2019b
Head and neck squamous cell carcinoma	*HOXA9*	-	93.8%	Promotes cancer progression and significantly correlated with lymph node metastasis	Zhou et al. 2019
Epithelial ovarian cancer	*HOXA9*	82.3%	88.6%	Potential diagnostic serum marker for early detection	Singh et al. 2020
Lung cancer	*HOXA9*	-	84.2%	Biomarker for lung cancer subtyping in liquid biopsies	Nunes et al. 2019
Colorectal cancer	*HOXD10*	-	-	Promotes cell proliferation, migration, and invasion	Yuan et al. 2019
	*HOXA2, HOXA5, HOXA6*	-	-	Significant association with age, stage, lymphovascular and perineural invasion	Li et al. 2019a
Cholangiocarcinoma	*HOXD9*	-	90%	A potential diagnostic marker: Promotes dedifferentiation of cholangiocytes	Wasenang et al. 2019
*Non-coding RNAs and chromatin modification*					
LncRNA-mediated *HOX* gene regulation					
Cancer	HOX-embedded lncRNA	Interaction with chromatin modifiers	Target, *HOX* genes	Role	References
Breast cancer, glioblastoma multiforme	HOTAIRM1**↑**	EZH2	*HOXA1***↑**	Promotes resistance to tamoxifen; promotes cell proliferation, migration, and invasion and suppresses apoptosis	Kim et al. 2020; Li et al. 2018
Prostate cancer, pancreatic cancer, and hepatocellular carcinoma	HOTTIP**↑**	WDR5	*HOXA9*, *HOXA13***↑**	Promotes tumorigenesis	Fu et al. 2017; Malek et al. 2017; Quagliata et al. 2014
Most of the cancer types	HOTAIR**↑**	PRC2, LSD1	HOXD cluster genes↓	Promotes cell cycle progression, migration, EMT, and metastasis	Bhan and Mandal 2015
Colorectal cancer	HOXD-AS1↓	PRC2	*HOXD3*↓	Promotes proliferation, migration, and metastasis	Yang et al. 2019
miRNA-mediated *HOX* gene regulation					
Cancer	HOX-embedded miRNA	Target, *HOX* genes	Downstream targets	Role	References
Ovarian cancer	miR-10b↑	*HOXD10*↓	*MMP14*, *RHOC*	Induces migration and invasion; contributes to the metastatic phenotype	Nakayama et al. 2013
	miR-196b**↑**	*HOXA9*↓	*-*	Induces cancer cell invasion and recurrence	Chong et al. 2017
Non-small cell lung cancer	miR-196a**↑**	*HOXA5*↓	*-*	Promotes cell proliferation, migration, and invasion	Liu et al. 2012
Pancreatic cancer	miR-10a**↑**	*HOXA1*↓	*-*	Promotes invasive potential	Ohuchida et al. 2012
	miR-10a**↑**	*HOXB1 and HOXB3*↓	*-*	Promotes metastatic behavior	Weiss et al. 2009
Glioma	miR-10b**↑**	*HOXB3*↓	*HMGB1*, *RHOC*, and *MMP2*	Promotes cell proliferation, migration, and invasion	Li et al. 2019c
Endometrial cancer	miR-10b**↑**	*HOXB3*↓	*-*	Promotes cell proliferation, migration, and invasion	Chen et al. 2016
Clear-cell renal cell carcinoma	miR-10b↓	*HOXA3***↑**	*FAK*, *YAP*	Promotes cell invasion and metastasis	He et al. 2019

NOTE:YAP, Yes-associated protein; FAK, focal adhesion kinase; HOTAIR-HOX, transcript antisense RNA; HOTAIRM1-HOX, antisense intergenic RNA myeloid 1; HOXD-AS1-HOXD, cluster antisense RNA 1; HOTTIP-HOXA, distal transcript antisense RNA; EZH2, enhancer of zeste 2; PRC2, polycomb repressive complex 2; WDR5-WD, repeat domain 5; LSD1, lysine-specific histone demethylase 1A; HMGB1, high mobility group box protein 1; **↑**, upregulated; ↓, downregulated

Further, in cervical cancer (CC), the methylation of the first exon of the *HOXA9* gene resulted in gene repression. Ectopic expression of the *HOXA9* gene significantly induced an epithelial phenotype and suppressed cell proliferation and migration (Alvarado-Ruiz et al. 2016). Hence, understanding the dynamics of methylation and the discovery of novel epigenetic drugs might be a promising strategy to treat cancer.

#### ncRNAs and chromatin modification

One of the integral components of epigenetic machinery regulating gene expression at the transcriptional and post-transcriptional level is the ncRNA. However, chromatin modification, DNA methylation, and ncRNAs operate in a common mechanism of gene regulation. Botti et al. (2018) have extensively reviewed the dynamic role of HOX-embedded ncRNAs such as long non-coding RNAs (lncRNAs) and microRNAs (miRNAs) in regulating the *HOX* gene expression in different cancers (Botti et al. 2018). There are several highly conserved lncRNAs transcribed from specific *HOX* gene clusters. They control *HOX* gene expression in *cis* or *trans* by binding to chromatin modifiers or by sponging miRNAs (Rinn et al. 2007). The lncRNAs interact with chromatin remodeling complexes having specific enzymatic activity such as methyltransferases, demethylases, acetyltransferases, and deacetylases recruiting them to specific gene loci. Based on the complexes that are recruited to the gene loci, the target genes are either expressed or repressed. The repressing methylation marks (activating acetylation marks) at H3K27 and H3K9 positions and the activating methylation mark at H3K4 position on histone tails determine the gene expression pattern. The roles of HOX-embedded lncRNAs in regulating HOX cluster genes and their implications in the biological responses have been studied in different cancer types (Table 2).

There are six widely studied miRNAs embedded in the four HOX clusters that belong to 3 miRNA families, namely, miR-196, miR-10, and miR-615 (Tanzer et al. 2005). The impact of deregulated HOX-embedded miRNAs on *HOX* gene expression in different kinds of cancers has been summarized in Table 2. They bind to the 3′UTR of mRNA and facilitate transcriptional inactivation of tumor suppressor genes. Considering several genetic and epigenetic factors contribute to the aberrant expression of *HOX* genes in cancer, their gene expression profiling is crucial for clinical applications.

## Role of HOX-targeted signaling in metastatic progression

**Fig. 3:**
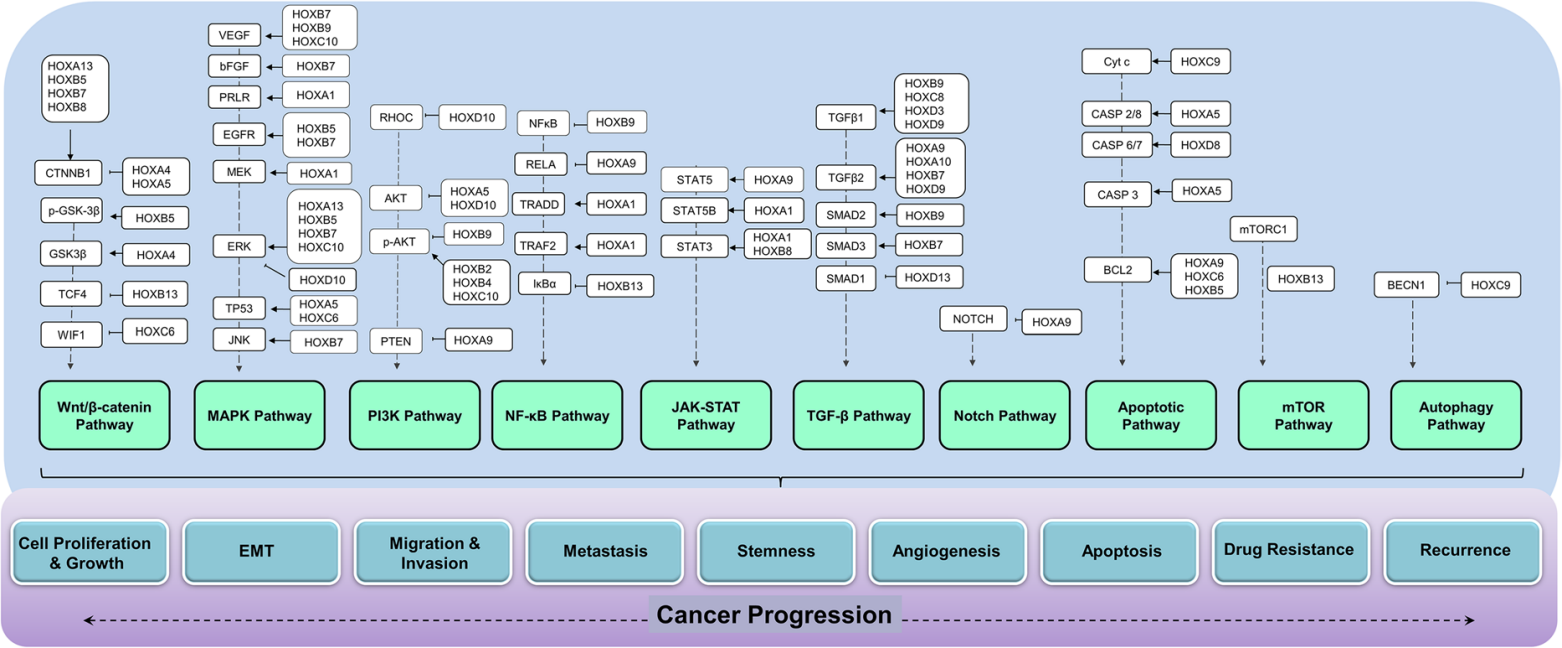
Aberrant regulation of *HOX* genes causing an imbalance in the key signaling pathways leading to metastatic progression in cancer

“Metastasis” reflects the critical step of tumor progression in which the cancer cells disseminate from the circulation into the surrounding tissues to form a secondary tumor (Hanahan and Weinberg 2011; Sever and Brugge 2015). The progression to metastasis requires the cancer cells to transform into mesenchymal phenotype, leave the primary site, acquire migratory capacity, circulate and survive in the bloodstream, evade apoptosis, disseminate to the distant site, acclimate to the new site, and generate new blood vessels for their further growth. The occurrence of these cellular processes is due to the imbalance in the important signal transduction pathways (Sever and Brugge 2015). Studies have found that HOX transcription factors regulate a large number of genes that are dynamically involved in the regulation of downstream signaling molecules (Rezsohazy et al. 2015). They preserve the integrity of the cells by regulating the diverse biological processes and functional pathways (Hombría and Lovegrove 2003; Moens and Selleri 2006). Hence, the deregulation of HOX proteins is directly linked to the imbalance in the key signaling pathways, which ultimately trigger the metastatic progression (Fig. 3). In the following section, we have discussed the molecular mechanism of HOX protein-mediated signaling pathways associated with the metastatic progression in cancer.

### Cell proliferation

The proliferative signal due to the prolonged activation of multiple signaling pathways is the first important property acquired by the cancer cells. Aberrantly expressed HOX proteins activate cancer cell proliferation by targeting multiple signaling pathways. The downregulated HOXA5 protein induces cell proliferation and neoplastic progression in CC via modulating the Wnt/β-catenin signaling and AKT pathway (Ma et al. 2020; Wang et al. 2019c). Gene set enrichment analysis and the TOP/FOP reporter assay confirmed that ectopic expression of *HOXA5* suppresses *CCND1* (cyclin D1) gene expression by inhibiting the nuclear translocation of β-catenin. It inhibits cell proliferation by upregulating the cell cycle inhibitor, p21 (*CDKN1A*), via transactivating *TP53*. The transactivation refers to the binding of HOXA5 to the 5′-TAAT-3′ motif of the *TP53* promoter through its HD domain (Ma et al. 2020). Further, HOXA5 inhibits cell proliferation also by upregulating the p27 gene (*CDKN1B*) via controlling the phosphorylation of AKT (Wang et al. 2019c). Thus, HOXA5 has the potential to inhibit cancer stem cell proliferation and metastasis.

Ordóñez-Morán et al. (2015) found that *HOXA5* is downregulated in CRC and its re-expression induces loss of cancer cell phenotype. They identified the presence of mutual antagonistic relation between *HOXA5* and Wnt signaling. Wnt signaling represses *HOXA5* to maintain stemness, whereas HOXA5 gets activated outside the intestinal crypt to suppress Wnt signaling and induce differentiation (Ordóñez-Morán et al. 2015).

In gastric cancer (GC), sustained proliferation is maintained by the activation of the HOXA13-induced Wnt/β-catenin signaling pathway. HOXA13 protein binds to the promoter region of the *CDH17* gene (cadherin-17), a downstream effector of HOXA13. Further, knockdown studies confirmed that *HOXA13* and *CDH17* could also promote cell invasion and inhibit apoptosis in GC cell lines (Qu et al. 2017). The aberrant proliferation of cancer cells is due to the disruption of T cell factor-4 (TCF-4)-stimulated Wnt/β-catenin signaling pathway (Angus-Hill et al. 2011; Arce et al. 2006). There is evidence that targeting TCF-4 could restrain the progression of cancer (Shin et al. 2017). Interestingly, overexpressing *HOXB13* in CRC caused growth suppression by inhibiting TCF-4 and its target *c-myc* at their translational level (Jung et al. 2005). Similarly, gain-of-function experiments demonstrated that HOXB13 functions as a suppressor of TCF-4 and its responsive genes (*c-myc* and *CCND1*) in PCa. This study demonstrated that HOXB13 could suppress the promoter activity of the *c-myc* gene and negatively regulate cell proliferation (Jung et al. 2004).

*HOXB13* overexpression has been implicated in drug resistance in BC cells. Elevated HOXB13 confers resistance to tamoxifen by downregulating the protein expression of estrogen receptor-α (ER-α) in ER + BC cells. HOXB13 is involved in both estrogen-independent and mammalian target of rapamycin (mTOR)-dependent proliferation of ER + BC cells. HOXB13 driven interleukin-6 (*IL-6*) expression, and subsequently, IL-6-mediated activation of mTOR/AKT pathway is another mechanism of BC cell proliferation and fibroblast recruitment (Shah et al. 2013).

In addition, elevated expression of HOXB7 protein confers resistance to tamoxifen in BC cells via the activation of the epidermal growth factor receptor (EGFR) pathway (Jin et al. 2012). EGFR, a receptor tyrosine kinase (RTK), is considered as a biomarker for tumor chemoresistance (Montagut et al. 2012; Wei et al. 2013). Inappropriate activation of EGFR either by transcriptional upregulation or by ligand overactivation is the most common event in oncogenesis (Sigismund et al. 2018). In BC, *HOXB7* functions as an ERα-responsive gene, which binds directly to the promoter region of *EGFR* to facilitate transcriptional activation. Hence, the therapeutic resistance of BC cells is linked to the HOXB7-induced crosstalk between RTKs and ERα-signaling (Jin et al. 2012).

Prolonged activation of nuclear factor kappa-light-chain-enhancer of activated B cells (NF-κB) signaling has been shown to induce tumor proliferation and cell survival in solid tumors (Sau et al. 2016; Xia et al. 2018). The non-canonical NF-κB pathway is activated by tumor necrosis factor-α (TNF-α) that binds to the tumor necrosis factor receptor (TNFR) family members (Cildir et al. 2016). Upon ligand-dependent activation, they multimerize and interact with tumor necrosis factor receptor type 1-associated death domain protein (TRADD) proteins to facilitate tumor necrosis factor receptor-associated factor (TRAF) protein-mediated activation of NF-κB signaling (Cildir et al. 2016). The activation of NF-κB signaling is critical for the oncogenicity of *HOXA1* in BC, which is functionally linked to the TNF/NF-κB signaling pathway. This study depicted a unique mechanism in which HOXA1 could activate TNF/NF-κB signaling pathway either by TRAF2 activation or by TRADD complex stabilization (Taminiau et al. 2016). This complex molecular interaction resulted in enhanced cell proliferation and loss of cell adhesion of BC cells.

Elevated *HOXB7* has been demonstrated in the peripheral lymphocytes in ALL patients whose progression is attributable to the HOXB7-mediated activation of basic fibroblast growth factor (bFGF) and extracellular signal-regulated kinase (p-ERK1/2) and inactivation of p27 (Zhong et al. 2019). Liao et al. (2011) demonstrated that the overexpression of *HOXB7* enhances growth and proliferation of CRC cells through the overactivation of the phosphatidylinositol 3-kinase (PI3K/AKT) and mitogen-activated protein kinase (MAPK) pathways. HOXB7 protein could also induce the expression of *CCND1* and repress the expression of *CDKN1B*, leading to the acceleration of G0-G1 to S-phase transition (Liao et al. 2011). Likewise, the HOXB8-mediated Wnt/β-catenin signaling pathway has been implicated in CRC proliferation and migration. The overexpression of Wnt signaling–related genes and their downstream targets such as matrix metalloproteinase-2 (*MMP2*), *c-Myc*, *CCND1*, and vimentin (*VIM*) indicates the involvement of Wnt/β-catenin signaling in *HOXB8-*overexpressed CRC cells (Li et al. 2019e).

Interestingly, in vivo studies demonstrated that upregulated HOXC6 could inhibit the Wnt inhibitory factor-1 (WIF-1) expression and activate Wnt signaling pathway in glioma cells. Through xenograft assay, immunohistochemistry (IHC), and knockdown studies, it was shown that knockdown of *HOXC6* could significantly block the cell cycle progression of U87 cells at G0/G1 phase and inhibit the colony formation. Hence, *HOXC6* could be a suitable target for clinical therapy (Yan et al. 2018).

MAPK pathway is the most common oncogenic pathway that involves sequential coordination among RTKs, growth factor receptor binding protein-2 (GRB2), and Ras/Raf/MEK/ERK signaling molecules to drive cancer progression (Guo et al. 2020b). The key molecules of the MAPK pathway such as GRB2 and MEK1 are reported as the downstream targets of HOXA1. HOXA1 induces cell proliferation, survival, and oncogenic transformation of human mammary epithelial cells by increasing the expression of GRB2/MEK1 and facilitating the p44/42 MAPK phosphorylation (Mohankumar et al. 2007). HOXA1-mediated upregulation of PRLR (prolactin receptors) and their subsequent signal transduction is another important mechanism demonstrated in the oncogenic transformation of mammary carcinoma cells (Hou et al. 2012).

HOXA1 also regulates the signal transducer and activator of transcription (STAT) signaling pathway to facilitate the oncogenic transformation of immortalized human mammary epithelial cells (Mohankumar et al. 2008). Aberrant activation of the Janus tyrosine kinase (JAK)/STAT pathway is chiefly linked to tumor progression. Higher levels of STAT3 and STAT5 protein expression and their phosphorylation have shown to be associated with poor survival and recurrence in solid tumors (Thomas et al. 2015). In human mammary carcinoma, HOXA1 could enhance the expression and phosphorylation of STAT3 and STAT5B to promote cell survival and proliferation (Mohankumar et al. 2008).

de Bock et al. in 2018 demonstrated that *HOXA9* functions as an oncogene in T-ALL and provides a platform for the JAK/STAT pathway activation and drive leukemia development. Transcriptional regulation of *STAT5* by HOXA9 was further confirmed by RNA-seq, chromatin immunoprecipitation sequencing (ChIP-seq), and assay for transposase-accessible chromatin sequencing (ATAC-seq). Further, the study found PIM1 kinase (proto-oncogene serine/threonine-protein kinase) as the target gene of both HOXA9 and STAT5 (de Bock et al. 2018). In esophageal squamous cell carcinoma (ESCC), the interplay of HOX proteins and the NOTCH signaling pathway is established. HOXA9*/*myeloid ecotropic viral insertion site 1 (MEIS1) complex suppresses NOTCH transcription factor, mastermind-like proteins (MAML1), and hence, the complex is considered as a negative regulator of the NOTCH pathway. The downregulation of MEIS1 in ESCC enhances the NOTCH pathway to drive invasion and cancer progression (Abbaszadegan and Moghbeli 2018).

In AML, overexpressed HOXA10 functions as an autocrine stimulator to increase the cell population of myeloid progenitor cells via the transforming growth factor-beta (TGF-β) signaling pathway (Shah et al. 2011). The binding of TGF-β ligands and phosphorylation of receptors, followed by the activation of small mothers against decapentaplegic SMAD) proteins, trigger this pathway (Syed 2016). HOXA10 protein upregulates the transcription of *TGFβ2* by interacting with tandem cis-elements in its promoter region and facilitate the expansion of hematopoietic stem cells and progenitor cells in AML (Shah et al. 2011).

### Epithelial–mesenchymal transition (EMT), migration, and invasion

Epithelial–mesenchymal transition involves loss of epithelial cell polarity, disruption of the cell to cell adhesion, remodeling of cytoskeleton, anchorage-independent growth, acquisition of mesenchymal phenotype, and gain of migratory and invasive properties (Kalluri and Weinberg 2009). EMT induces intrinsic signals in the cancer cells to confer tumor initiation potential and therapy resistance (Dongre and Weinberg 2019).

Upregulation of HOXB7 protein enhances EMT by increasing the expression of functional EMT proteins such as N-cadherin (*CDH2*) and vimentin in GC cells. The regulation of two critical members of MAPK pathways (ERK, p38α) by HOXB7 at their phosphorylation level contributes to the enhanced migratory and invasive properties of cancer cells. HOXB7 regulates the invasion of GC cells via AKT pathway members. The mechanism by which HOXB7 regulates phosphatase and tensin homolog (PTEN), PI3K, MKK3/6, and RTKs needs further exploration (He et al. 2017). Additionally, HOXB5 functions as an important regulator of Wnt/β-catenin signaling in GC. The upregulation of *HOXB5* in GC tissues significantly correlates with β-catenin (*CTNNB1)* gene expression. HOXB5 binds to the promoter region of *CTNNB1* and thus functions as a transcriptional activator. Using the overexpression and knockdown models, the study confirmed that *HOXB5* is involved in subsequent transcriptional activation of *CTNNB1* and downstream target genes such as *CCND1* and *c-Myc*, leading to the increased invasion and migration of GC cells (Hong et al. 2015).

The TGF-β pathway has been recognized as one of the most potent inducers of EMT (Xu et al. 2009). In GC, high expression of HOXD9 induces proliferation, migration, and invasion by controlling *TGFβ1* and *TGFβ2* expression (Xiong et al. 2020). Also, the overexpression of *HOXA13* has been associated with proliferation, migration, and invasion in GC. Besides, HOXA13 upregulates the expression of *CDH2* and *VIM* and downregulates the expression of *CDH1* and hence plays a critical role in EMT. RNA-Seq analysis revealed that *HOXA13* promotes GC progression by activation of *ERK1/2.* Hence, *HOXA13* could thus be a novel target for anticancer therapy in GC (Qin et al. 2019).

Some HOX factors are found to be involved in inhibiting EMT and promoting mesenchymal–epithelial transition (MET) in GC. Interestingly, HOXB9 in GC cells upregulates the *CDH1* expression by downregulating the N-cadherin and Snail (*SNAI1*) protein expression. Ectopic *HOXB9* could induce apoptosis by inhibiting AKT signaling pathway and blocking the expression of p-AKT, p-glycogen synthase kinase-beta (p-GSK3β), and NF-κB (Zhang et al. 2019). It was proposed that the interaction of HOXB9-PBX1 (Pre-B cell leukemia transcription factor 1) was responsible for GC growth. When HOXB9 protein gets uncoupled from oncoprotein PBX1, it could suppress p-AKT and NF-κB-dependent *SNAI1* expression to induce cell death and MET (Zhang et al. 2019). However, HOXB9 induces *SNAI1* and *SNAI2* (characteristic markers of EMT) and promotes invasion in oral squamous cell carcinoma (OSCC) cell lines by upregulating *TGFβ1/SMAD* genes (Xue et al. 2017). HOXB9 functions as a transcriptional activator and induces *TGFβ1* in hepatocellular carcinoma (HCC) cells, enhancing their migratory and invasive potential (Sha et al. 2015).

In head and neck squamous cell carcinoma (HNSCC), *HOXB5* functions as an oncogene, which binds directly to the promoter region of EGFR and regulates the AKT/Wnt/β-catenin synergistic signaling axis to enhance proliferation, cell migration, invasion, and EMT (Lee et al. 2020). In BC, HOXB5 is involved in the transcriptional activation of EGFR and its downstream targets to facilitate BC invasion (Lee et al. 2018). In BC and non-small cell lung cancer (NSCLC), knockdown of *HOXB5* significantly suppressed the β-catenin protein and its downstream targets *c-Myc* and *CCND1*, thereby inhibiting cell proliferation, migration, invasion, and EMT of the cancer cells (Zhang et al. 2018a; Zhang et al. 2020). The anchorage-independent growth, proliferation, and migration property of NSCLC are also induced by HOXC8 through the transcriptional activation of *TGFβ1*. Further, the overexpression of *HOXC8* results in chemoresistance to cisplatin. Targeting *HOXC8* is thus a suitable therapeutic approach for the chemo-sensitization of NSCLC cells (Liu et al. 2018).

Overexpressed HOXB5 induces EMT in pancreatic cancer (PC) via the Wnt/β-catenin signaling pathway. HOXB5 inherently activates the phosphorylation of GSK-3β at Ser-9 position and facilitates the nuclear accumulation of β-catenin. HOXB5 protein upregulates the expression of mesenchymal markers such as *VIM*, *SNAI1*, and *SNAI2* and downregulates the expression of *CDH1*, to enhance the mesenchymal transition, motility, and migratory ability of cancer cells (Gao et al. 2020). Similarly, HOXB7 activates ERK1/2 in PC to activate the downstream genes and induce the motility and invasion of cancer cells (Tsuboi et al. 2017). IHC analysis showed the localization of HOXB7 in the cell protrusions of migrating PC cells. HOXB7 could induce the phosphorylation of ERK1/2 and thereby promotes the cell protrusions to enhance the invasive potential. The invasiveness is also due to the HOXB7/ERK1/2-mediated stimulation of c-Jun N-terminal kinases (JNK) and phosphorylation of small heat shock protein (sHSP27) (Tsuboi et al. 2017). In addition to this, overexpressed HOXA10 also promotes invasiveness of PC cells via the TGFβ2-p38 MAPK pathway. It upregulates the level of *TGFβ2* and *MMP3* and activates p38 in PC cells to promote migration and invasion (Cui et al. 2014).

The cleavage of extracellular matrix (ECM), detachment of cancer cells from the basement membrane, and anchorage-independent growth followed by their migration and invasion are mainly due to the activity of several important proteases. In retinoblastoma (RBM), HOXB5 significantly contributes to the migration and invasion of cancer cells by stimulating the activation of the ERK1/2 pathway and subsequent production of the proteases MMP-3 and MMP-13 (Xu et al. 2018). Studies have demonstrated that aberrantly expressed *HOX* genes confer a growth advantage in glioblastoma (GBM). For example, HOXC10 promotes EMT by activating the PI3K pathway and inducing the expression of p-PI3K and p-AKT (Guan et al. 2019). Further, the HOXB2-induced PI3K pathway has been considered a potential therapeutic target in GBM. The knockdown of *HOXB2* in GBM cell lines resulted in a reduction of proliferation and invasion via the PI3K/AKT pathway. The involvement of the PI3K pathway was confirmed by the increased levels of p-PI3K and p-AKT upon *HOXB2* overexpression (Li et al. 2020c). It has also been shown that the overexpression of *HOXC9* contributes to tumorigenicity in GBM. It suppresses the autophagy pathway by transcriptionally inhibiting its downstream target gene, death-associated protein kinase 1 (*DAPK1*), resulting in the inhibition of the DAPK1/Beclin pathway (Xuan et al. 2016). Higher levels of HOXA13 expression promote migration, invasion, and EMT of GBM cells by the activation of Wnt/β-catenin and TGF-β pathways. The small interfering RNA (siRNA)-mediated *HOXA13* silencing prevents the nuclear localization of β-catenin, correlating significantly with decreased migration and invasion. Elevated levels of HOXA13 induce EMT with the stabilization of p-R-SMAD in the nucleus and activation of the TGF-β pathway (Duan et al. 2015). In another study, it was shown that the elevated HOXC6 in GBM significantly induces the expression of MAPK pathway genes including *c-Myc*, *c-Jun*, *TP53*, JNK, ERK, and P38 genes, promoting cell proliferation and migration (Yang et al. 2019b).

HOXD10 is downregulated in both cholangiocellular carcinoma (CCC) and CRC. Decreased HOXD10 levels mediate the activation of Ras homolog gene family, member C (RHOC), and MMPs and thus confer prolonged invasiveness and decreased apoptosis. The overexpression of *HOXD10* deactivates RHOC/AKT/MAPK pathway to constrain cell migration and invasion and promote apoptosis (Yang et al. 2015; Yuan et al. 2019). The downregulation of HOXD10 is also shown to be associated with cell proliferation, cell cycle arrest, colony formation, migration, invasion, and apoptosis of HCC cells through the activation of the ERK signaling pathway (Guo et al. 2017b). Similarly, HOXA5 is underexpressed in both the CC tissues and cell lines, and its overexpression inhibits tumor progression by regulating AKT/p27 activation (Wang et al. 2019c).

### Evasion of apoptosis

Following cancer cell invasion events such as intravasation, survival of tumor cells in the circulation, and resistance to cell death by immune surveillance evasion precede distant metastasis (Chiang et al. 2016). Aberrant regulation of apoptosis noted in cancer cells is due to altered homeostasis to thrive in the cellular environment (Jan and Chaudhry 2019).

Over the past decades, researchers have been extensively studying the role of HOX proteins in the regulation of key molecules of the apoptosis pathway in different cancers. They either enhance the activation of the caspase family of proteins or induce the transcription of pro-apoptotic/anti-apoptotic proteins, thereby activating two distinct and converging pathways (intrinsic and extrinsic) of apoptosis.

Aberrantly expressed HOX proteins have an inherent ability to activate the transcription of the anti-apoptotic gene B cell lymphoma 2 (*BCL2*) in different cancer types. In HCC, overexpressed HOXB5 inhibits apoptosis by increasing the protein levels of BCL-2 and decreasing the pro-apoptotic proteins Cyt c, BAX, and caspase-3. Further, it was demonstrated that HOXB5 could induce the expression of murine double minute 2 oncogene (*MDM2*) in hepatoma cells through ERK/MDM2 signaling (Su et al. 2020). In CC, elevated HOXC6 functions as a positive regulator of anti-apoptosis. However, *HOXC6* knockdown results in the downregulation of *BCL2* to activate the intrinsic apoptosis pathway (Wang et al. 2019b). The regulation of the apoptosis pathway by *HOXC6* has also been reported in HNSCC. HOXC6 could bind to the TAAT motif of the *BCL2* promoter region located − 420 bp upstream to TSS to transcriptionally upregulate its expression. The inherent anti-apoptotic property of *HOXC6* is the reason for resistance to paclitaxel-induced apoptosis in HNSCC (Moon et al. 2012). A molecular link between *HOXA9* and *BCL2* has been established in AML. HOXA9 regulates *BCL2* expression to facilitate the survival of myeloid progenitors and the immortalization of hematopoietic cells (Brumatti et al. 2013). The role of HOXA9 in influencing the tumor microenvironment has been demonstrated in ovarian cancer (OVC). The overexpressed HOXA9 activates *TGFβ2* and promotes the transformation of normal peritoneal fibroblasts and normal adipose and bone marrow–derived mesenchymal stem cells (MSCs) into cancer-associated fibroblasts (CAFs) (Ko et al. 2012). In contrast to this observation, HOXA9 was significantly downregulated in cutaneous squamous cell carcinoma (CSCC). The downregulation of HOXA9 facilitates the activation of the p65-subunit of NF-κB (*RELA*) to activate the NF-κB pathway. The synergistic action of NF-κB, apoptosis, and autophagy pathways promotes CSCC progression. HOXA9-mediated activation of NF-κB signaling has promoted anti-apoptosis through BCL-extra large (BCL-XL) and repressed autophagy by regulating autophagy-related genes (ATG) such as *ATG1*, *ATG3*, and *ATG12* (Han et al. 2019).

In GBM, the overactivation of HOXA9 is significantly correlated with the inactivation of the negative regulator of the PI3K pathway (PTEN), enhancing cell proliferation and suppressing apoptosis. However, HOXA9 inhibits apoptosis by diminishing tumor necrosis factor-related apoptosis-including ligand (TRAIL) in the GBM cell lines. Hence, reversing *HOXA9* activation by PI3K inhibition may be of therapeutic significance in human GBM (Costa et al. 2010).

In BC, HOXA5 functions as a key molecule in the regulation of both p53-independent and p53-dependent apoptotic pathways (Raman et al. 2000). The study demonstrated that the p53 promoter region is enriched in HOXA5 binding sites, and hence, it regulates the endogenous synthesis of p53. Compromised *HOXA5* function due to promoter methylation could lead to the loss of p53 expression in human BC (Raman et al. 2000). Further, HOXA5 could regulate the p53-independent extrinsic apoptosis pathway in BC and sensitize the cancer cells to TNF-α-induced apoptosis by regulating caspases 2 and 8 (*CASP2/CASP8*) (Chen et al. 2004). Likewise, HOXA5 enhances apoptosis by inducing caspase-3 (*CASP3*) activity in CC. The gain-of-function of *HOXA5* in CC cell lines confirmed that HOXA5 could act as a tumor suppressor by inducing the expression of cell cycle inhibitor p27 and suppressing the phosphorylation of AKT (Wang et al. 2019c). Re-expression of *HOXA5* could also induce apoptosis of osteosarcoma cells by inducing *CASP3* activity and promoting the p53-dependent p38α MAPK pathway (Chen et al. 2019). Similarly, *HOXD8* exerts a tumor suppressor role in CRC. The stable *HOXD8* expression in CRC cell lines upregulated the expression of executioner caspase-6 and caspase-7 (*CASP6/CASP7*) and cleaved poly(ADP-ribose) polymerase (PARP) to induce apoptosis in CRC cells (Mansour and Senga 2017). In infant neuroblastoma, HOXC9 significantly triggers the intrinsic apoptosis pathway via releasing cytochrome c (Cyt c) into the cytosol. The activation of apoptosis pathway following increased expression levels of HOXC9 was significantly correlated with spontaneous regression in infant neuroblastoma (Kocak et al. 2013).

### Metastasis

The dissemination of cancer cells to the surrounding tissues is facilitated by the activation of several key molecules (Fares et al. 2020). Deregulated *HOX* genes significantly induce distant metastasis by targeting key signaling pathways. A study on HCC demonstrated that *HOXB7* is highly expressed in cells with high metastatic potential. Further, microarray data of 394 HCC tissues confirmed that *HOXB7* modulates the biological functions of cancer cells via the activation of the MAPK/ERK pathway. Overexpressed HOXB7 binds to the promoter region of *bFGF* and induces *bFGF* secretion. Thus, *HOXB7* functions as a molecular signature for predicting prognosis, survival, and recurrence (Wang et al. 2017). The elevated *HOXB7* in lung adenocarcinoma (LAC) is linked to enhanced lymph node metastasis through the TGF-β/SMAD3 signaling pathway (Zhuang et al. 2015). The clinical relevance of the HOXB7-mediated TGF-β signaling pathway in BC demonstrated that *HOXB7* activates *TGFβ2* transcription by binding directly to its promoter region. Loss of HOXB7-induced *TGFβ2* in BC cell lines has significantly reduced lung metastasis. The overexpression of HOXB7 promotes metastasis by inducing tumor-associated macrophage (TAM) recruitment (Liu et al. 2015a). While investigating the molecular mechanism of BC recurrence and distant metastasis after chemotherapy, researchers found that HOXC10 was responsible for suppressing apoptosis and inducing the NF-κB pathway in the BC cells treated with doxorubicin, paclitaxel, or carboplatin. Further, a unique and complex mechanism of HOXC10-mediated S-phase specific DNA damage repair was noted in the BC cells. Besides facilitating the recruitment of homologous recombination proteins (HR) to the site of DNA damage, HOXC10 reinitiates DNA replication by resolving stalled replication forks. Moreover, it could also resume RNA polymerase-II-dependent transcription by binding to the cyclin-dependent kinase 7 (*CDK7*). Hence, blocking the expression of *HOXC10* in BC could be a useful therapeutic strategy (Sadik et al. 2016).

Researchers have determined the role of *HOXB8* in CRC in promoting EMT via STAT-3 activation. The induction of EMT was characterized by the presence of mesenchymal markers such as *VIM*, *CDH2*, *TWIST*, *ZEB1*, and *ZEB2*. Interestingly, HOXB8 not only promotes lung metastasis but also enhances lymphatic metastasis in CRC (Wang et al. 2019a). Also, HOXB8 contributes to osteosarcoma progression and metastasis by activating the Wnt/β-catenin signaling pathway (Guo et al. 2019).

Decreased intracellular zinc concentration is the common characteristic of prostate malignancy (Huang et al. 2006). HOXB13 promotes metastasis of PCa by decreasing intracellular zinc through the NF-κB pathway. The activation of the NF-κB pathway may be due to the HOXB13-regulated nuclear localization of p65 transcription factors by reducing the levels of the NF-κB inhibitor, IκBα (Kim et al. 2014). HOXA13 and HOXC10 elevation significantly activate the MAPK pathway to induce proliferation and metastasis in GC (Guo et al. 2017a; Qin et al. 2019). However, the migration and invasion were due to the HOXA13-mediated activation of ERK1/2 and HOXC10-mediated upregulation of MAPK signaling–related genes (Guo et al. 2017a; Qin et al. 2019).

Meanwhile, *HOXD3* expression is associated with an increase in the anchorage-independent growth, proliferation, and migration of NSCLC. Elevated HOXD3 reduces the expression of TGF-β-independent tumor-suppressing genes (desmoglein, desmoplakin, and plakoglobin) and upregulates the expression of TGF-β induced tumor-related genes such as *MMP2*, syndecan-1 (*SDC1*), and *CD44*. Thus, HOXD3 promotes the invasive and metastatic potential of cancer cells through the TGF-β-dependent and TGF-β-independent pathways (Miyazaki et al. 2002). Further, detailed information regarding the metastatic progression regulated by HOX proteins has been summarized in Table 3.

**Table 3:** Contribution of *HOX* genes in cancer invasion and metastasis

Cancer	Deregulated *HOX* genes	Signaling pathway	Targets	Role	References
Gastric cancer	**↑** *HOXC10*	**↑** NF-κB	*ATM*	Promotes cell migration and invasion; significantly associated with TNM stage, lymph node metastasis, and distant metastasis	Yao et al. 2018
Cutaneous squamous cell carcinoma	**↑** *HOXB7*	**↑** Wnt/β-catenin	*CTNNB1*	Enhances cell viability, migration, invasion, and metastasis and inhibits apoptosis	Gao and Chen 2018
Osteosarcoma	**↑** *HOXB8*	**↑** Wnt/β-catenin	*CTNNB1*, *c-Myc*, *CCND1*	Promotes proliferation, migration, invasion, and metastasis	Guo et al. 2019
Breast cancer	**↑** *HOXB7*	**↑** TGF-β	*TGFβ2*	Induces metastasis via TAM recruitment; promotes tumor progression in a cell-autonomous and non-cell-autonomous manner; and enhances migration and invasion	Liu et al. 2015
	**↑** *HOXC10*	**↑**NF-κB	*-*	Significantly correlated with distant metastasis after chemotherapy	Sadik et al. 2016
Hepatocellular carcinoma	**↑** *HOXB5*	**↑** TGF-β-induced PI3K/AKT	*-*	Promotes cell migration and metastasis	(Sun et al. 2018)
Lung adenocarcinoma	**↑** *HOXB7*	**↑**TGF-β/SMAD3	*VEGFA*, *MMP2*, *SMAD3*	Significantly associated with lymph node metastasis	Zhuang et al. 2015
Prostate cancer	**↑** *HOXA1*	**↑**ERK1/2 and AKT	*ERK1/2*, *AKT*	Promotes cell proliferation, invasion, and metastasis	Wang et al. 2015
	↓ *HOXD13*	**↑** TGF-β/BMP4/SMAD1	*SMAD1*	Promotes EMT and metastasis	Xu et al. 2021
Lung cancer	↓ *HOXA4*	**↑** Wnt/β-catenin	*GSK3β*; β-catenin, cyclin D1, c-Myc, and survivin	Promotes proliferation, migration, invasion, and lymph node metastasis and inhibits cell cycle arrest	Cheng et al. 2018

NOTE: TAM, tumor-associated macrophage; ATM, ataxia telangiectasia mutated; **↑**, upregulated; ↓, downregulated

### Angiogenesis

The metastasis of cancer cells depends mainly on the adequate supply of oxygen which can be achieved by the formation of new blood vessels (Nishida et al. 2006). HOX proteins act as an activator of angiogenic factors to bring out the aggressive phenotype of cancer cells. In BC, Carè et al. (2001) demonstrated the mechanism of stimulation of tumor invasion and neo-angiogenesis by the elevated HOXB7 levels. HOXB7 stimulates angiogenic response by inducing the important pro-angiogenic factors such as bFGF, vascular endothelial growth factor (VEGF), melanoma growth-stimulatory activity/growth-related oncogene alpha (MGSA/GRO-α), interleukin-8 (IL-8), and angiopoietin-2 (Ang-2) (Carè et al. 2001). Overexpressed HOXB9 has been involved in the stimulation of angiogenesis in high-grade BC. The pro-angiogenic response was confirmed by the induction of the expression of angiogenic molecules such as VEGF, bFGF, IL-8, and angiopoietin-like protein 2 (ANGPTL-2) upon *HOXB9* overexpression. There are multiple HOX-binding sites located in these factors, indicating their activation by HOXB9. Thus, HOXB9 enriches the tumor microenvironment with these angiogenic molecules, to facilitate vascularization of tumors and distant metastasis. In addition to this, HOXB9 overexpression stimulated EMT via the generation of spindle-shaped mesenchymal cells, detachment of cells from the matrix, and formation of actin filaments. The induction of EMT was correlated with the loss of *CDH1* and gain of *CDH2*, *VIM*, *SNAI1*, and *TWIST* markers (Hayashida et al. 2010).

In multiple myeloma, elevated HOXB7 expression contributed to angiogenesis by stimulating the expression of angiogenic factors. The upregulation of those angiogenic stimulators such as VEGFA, FGF2, MMP2, WNT5a, and platelet-derived growth factor subunit A (PDGFA) implicated in the blood vessel formation (Storti et al. 2011). In glioma, the vascular tube formation was enhanced by HOXC10 elevation. HOXC10 enhances the transcription of *VEGFA* by binding to its promoter region. Increased transcription and expression were followed by protein arginine methyltransferase 5 (PRMT5) and WD repeat domain 5 (WDR5)-mediated post-translational modification (Tan et al. 2018). Moreover, studies are required to better understand the role of HOX proteins in the regulation of angiogenesis in different types of cancer.

## Clinical significance of *HOX* genes in cancer

**Fig. 4:**
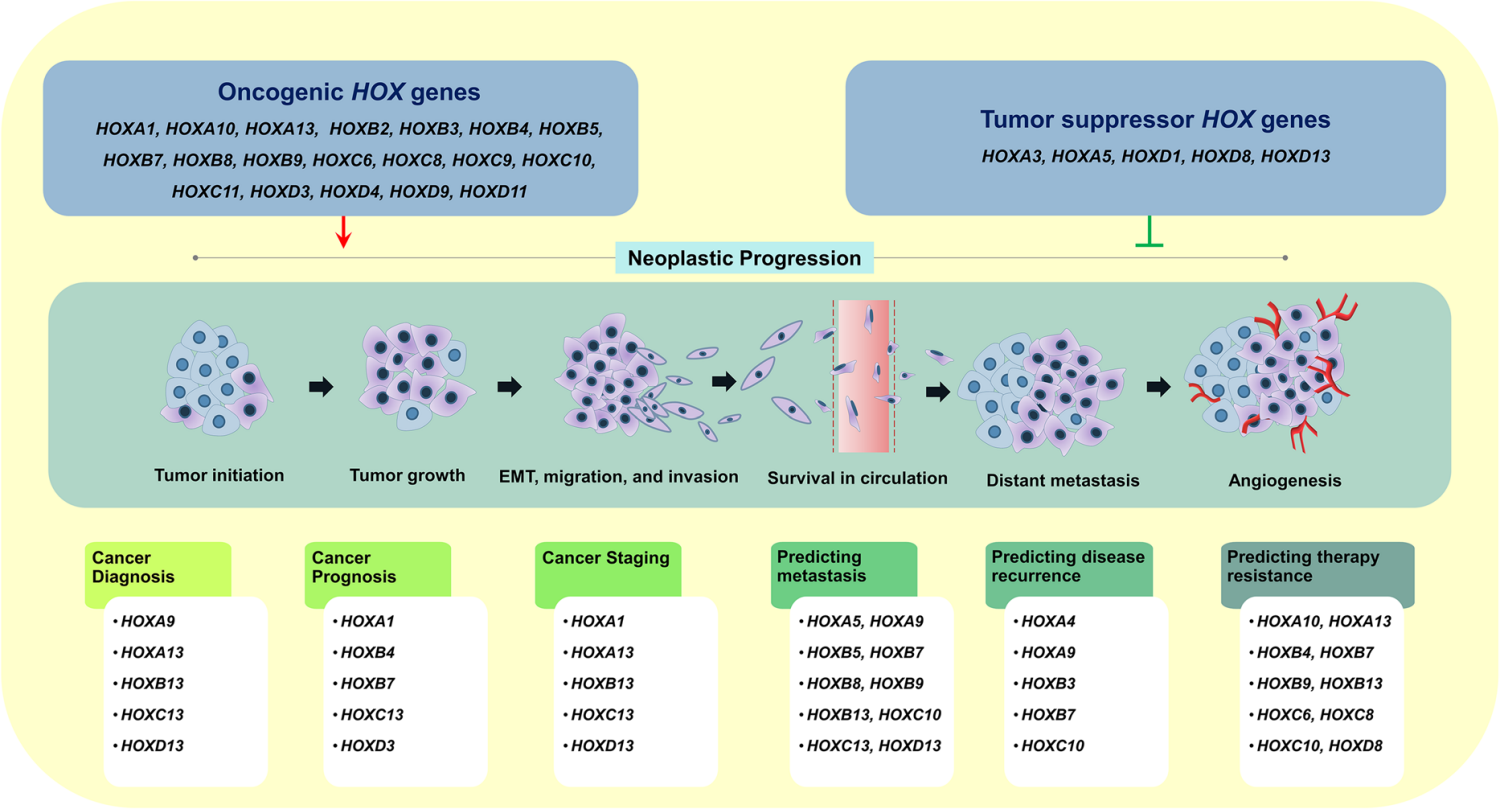
Oncogenic and tumor suppressor *HOX* genes in the neoplastic progression: their biological significance in cancer diagnosis, prognosis, and therapeutic response

Cancer is the major cause of death globally with the majority of patients present with advanced stages of malignancy due to the absence of reliable prognostic markers. Identifying the specific oncogenic molecule that induces metastatic progression could help clinicians to diagnose cancer, stratify the stages, and suggest a suitable treatment regimen for better clinical outcomes. Understanding the role of *HOX* genes as a potential biomarker in cancer and exploring the utility of HOX-associated targets in various therapies may alleviate the cancer burden and reduce mortality rates (Fig. 4). The clinical significance of *HOX* genes in different cancer types has been summarized in Table 4.

**Table 4:** Biological role and clinical implications of *HOX* genes in cancer

Deregulated *HOX* genes	Target	Cancer	Clinical Utility		References
*HOX* gene as a prognostic marker					
*HOXA5*↓	*CDKN1A*	Non-small cell lung cancer	Positively correlated with TNM staging		Zhang et al. 2015
*HOXA5, HOXA9*↑	*-*	Acute myeloid leukemia	Decreases the remission rate		Zhao et al. 2015
*HOXA10*↑	*MMP3*	Bladder cancer	Positively associated with pathological grade and clinical stage		Liu et al. 2019
*HOXA13*↑	*-*	Lung squamous cancer	Positively correlated with TNM stage		Zhang et al. 2017
*HOXA13*↑	*-*	Bladder cancer	Positively correlated with lymphatic metastasis, pathological grade, and TNM stage		Hu et al. 2017
*HOXB9*↑	-	Laryngeal squamous cell carcinoma	Positively correlated with histological grade		Sun et al. 2017
*HOXC6*↑	*Ki67*	Nasopharyngeal carcinoma	Significantly associated with tumor stage and advanced tumor status		Chang et al. 2017
*HOXC9*↑	-	Colorectal cancer	Positively correlated with tumor stage, distant metastasis, and venous invasion		Hu et al. 2019
*HOXC9*↑	-	Gastric cancer	Positively correlated with tumor size, lymphatic invasion, and lymph node metastasis		Zhao et al. 2020
*HOXC10*↑	-	Thyroid cancer	Positively correlated with advanced and poor pathological stage		Feng et al. 2015
*HOXC10*↑	*-*	Glioma	Positively associated high grading; induces immunosuppressive genes		Li et al. 2018
Role of *HOX* genes in therapy resistance					
*HOXA5*↑	-	Acute myeloid leukemia	Provides resistance to cytarabine	Li et al. 2015	
*HOXA9*↑	*BCL2*	Glioblastoma	Confers resistance to temozolomide	Pojo et al. 2015	
*HOXA10*↑	*PTEN*	Colorectal cancer	Provides resistance to 5-Fluorouracil	Yuan et al. 2018	
*HOXA11*↓	-	Glioblastoma	Confers resistance to radiation and temozolomidetherapy	Se et al. 2017	
*HOXA13*↑	-	Hepatocellular carcinoma	Provides resistance to sorafenib	Quagliata et al. 2018	
*HOXA13*↑	*MRP1*, *DHRS2*	Gastric cancer	Provides resistance to 5-Fluorouracil	Han et al. 2018	
HOXB cluster genes↑	-	Breast cancer	Provides tamoxifen resistancce	Yang et al. 2018	
*HOXB13*↑	ERα, *IL-6*	Breast cancer	Promotes tamoxifen resistancce	Shah et al. 2013	
*HOXC6*↑	*BCL2*	Head and neck squamous cell carcinoma	Confers paclitaxel therapy resistance	Moon et al. 2012	
*HOXC8*↑	*TGF-β1*	Non-small cell lung cancer	Provides cisplatin resistance	Liu et al. 2018	
*HOXC10↓*	-	Breast cancer	Confers resistance to aromatase inhibitors	Pathiraja et al. 2014	
*HOXD8*↑	-	Epithelial ovarian cancer	Promotes cisplatin therapy resistance	Sun et al. 2018	
*HOX* gene as a biomarker for predicting disease recurrence					
*HOXC10*↑	*-*	Gastric cancer	Positively associated with hepatic and peritoneal recurrence	Miwa et al. 2019	
*HOXA9*↓	-	Non-small cell lung cancer	Provides disease recurrence	Hwang et al. 2015	
*HOXA4/HOXB3*↑	-	High-grade serous ovarian carcinoma	Biomarker for recurrence after primary cytoreductive surgery and first-line adjuvant chemotherapy	Miller et al. 2018	
*HOXB7*↑	*bFGF*	Hepatocellular carcinoma	Provides disease recurrence	Wang et al. 2017	

NOTE: DHRS2, dehydrogenase reductase 2; CST1, cystatin SN; **↑**, upregulated; ↓, downregulated

### *HOX* gene as a diagnostic marker

To reduce the mortality of cancer and identify suitable therapy, accurate diagnosis is crucial. The existing literature supports the utility of *HOX* gene as a diagnostic marker. Sarno et al. (2020) established that the paralogous *HOX13* group of genes (*HOXA13*, *HOXB13*, *HOXC13*, and *HOXD13*) contributes to the progression of BC and aid in histological subtyping (Sarno et al. 2020). In childhood ALL, the overexpression of *HOXA9* and *MEIS1* showed a significant correlation with the ALL subtypes (Adamaki et al. 2015). The overexpression of HOXA13 was correlated with the grade, size, microvascular invasion, capsular spread in the nodes, alpha-fetoprotein (AFP) level, and tumor-node-metastasis (TNM) staging in HCC. *HOXA13*-expressing tumors showed shorter overall survival (OS) and disease-free survival (DFS) than those with *HOXA13*-negative tumors, indicating its role as a prognostic marker in HCC (Pan et al. 2014). In another study, the role of HOXA13 playing a crucial role in four glioma cell lines was established. Increased cell proliferation and invasion were associated with the activation of Wnt and TGF-β-induced EMT pathways indicating its role as a diagnostic marker (Duan et al. 2015).

### *HOX* gene as a prognostic marker

The transcriptome profiling and pathway analysis of *HOXA1* expressing melanoma cells showed increased TGF-β signaling. The potential role of HOXA1 in repressing genes required for melanocyte differentiation and activating pro-invasive genes via the TGF-β signaling pathway was distinctly demonstrated. The expression of *HOXA1* could stratify melanoma patients based on metastatic potential and hence function as a potential prognostic marker (Wardwell-Ozgo et al. 2014). Morgan et al. (2016) demonstrated the oncogenic nature of *HOXB4* in mouse models of mesothelioma. The overexpression of *HOXB4* was positively correlated with shorter OS (Morgan et al. 2016). Higher expression of *HOXB7* was detected in HCC tissues, which was found to be associated with a poor prognosis. The tumor-promoting and metastatic ability of *HOXB7* were validated in a xenograft tumor model and bioluminescence imaging, respectively. Interestingly, the stem cell markers such as epithelial cell adhesion molecule (*EPCAM*) and *NANOG1* (*Nanog*) were found to be upregulated upon HOXB7 overactivation (Huan et al. 2017).

An extensive bioinformatic analysis in BC revealed a positive correlation between *HOXC13* expression and lymph node metastasis, indicating a poor prognosis (Li et al. 2020a). Shaoqiang et al. (2013) revealed that BC patients with increased expression of *HOXD3* were significantly correlated with higher histopathological grade and shorter survival time (Shaoqiang et al. 2013). Taken together, these data reflect the possibility of using the HOX cluster as a potential prognostic marker for cancer progression.

### *HOX* gene as a biomarker for predicting metastasis

Cancer progression is mainly due to the ability of the neoplastic cells to metastasize to the distant sites of the body often via the bloodstream or lymphatics. The therapeutic goal should be the prevention of metastasis in advanced stages of malignancy. HOX-mediated invasion and metastasis in different cancer types have been reviewed recently (Paço et al. 2020a). Through the genome-wide transcriptomic and pathway analyses, Wang et al. (2015) showed that HOXA5 could suppress metastasis in NSCLC by interfering with the cytoskeleton remodeling mechanism (Wang et al. 2015a). Increased *HOXA9* expression in CRC tissues was shown to be associated with lymph node metastasis (Watanabe et al. 2018).

In another study, HOXB5 was shown to transactivate metastatic-related genes such as C-X-C motif chemokine receptor 4 (*CXCR4*) and integrin subunit beta 3 (*ITGB3*) promoter and propagate distant metastasis in CRC (Feng et al. 2021). The role of the HOXB7 in progression and metastasis in LAC confirms its prognostic potential and a suitable target for therapy (Yuan et al. 2014). Also, the metastatic ability of HOXB7 in HCC cells in vivo was confirmed with bioluminescence imaging. The study demonstrated how HOXB7 could induce stem-like properties and facilitate EMT in HCC (Huan et al. 2017). In CRC, *HOXB8* functioned as an oncogene whose expression was regulated by the MYC-super enhancer complex. HOXB8 has the potential ability to interact with metastatic regulator BACH1 (BTB domain and CNC homolog 1) and thereby potentiates the metastatic ability and invasive nature of cancer cells (Ying et al. 2020). For the first time, researchers demonstrated the ability of HOXB9 in promoting the metastatic nature of HCC cells. They found that the TGF-β pathway is the crucial signaling cascade in promoting EMT in HCC cells (Sha et al. 2015).

A study on OVC showed the critical role of HOXC10 in metastasis. HOXC10 could regulate the transcription of the EMT-related gene *SNAI2* and could induce metastatic ability (Peng et al. 2020). By inducing the metastasis-related genes such as 3-phosphoinositide-dependent protein kinase 1 (*PDPK1*) and vasodilator-stimulated phosphoprotein (*VASP*), HOXC10 promotes metastasis in HCC (Dang et al. 2020). The overexpression of *HOXC13* was observed in metastatic melanoma tissues when compared to primary melanoma tissues, implying its role as a metastatic inducer (Cantile et al. 2012).

Studies have hypothesized that targeting metastatic cancer and its related pathways might be a necessary approach for cancer therapy. As per the recent findings in PCa, HOXD13 is involved in inhibiting bone morphogenetic protein 4 (*BMP4*)*/SMAD1*-induced EMT and thus functions as a negative regulator of metastasis (Xu et al. 2021). Another study on PCa implicated *HOXB13* as a metastasis promoter gene that could induce metastasis via regulating mitotic protein kinases (Yao et al. 2019). Following radical prostatectomy surgery, tumors with high expression of *HOXB13* and a low expression of its binding partner *MEIS1/2* were significantly associated with an increased propensity for metastasis (Weiner et al. 2020). These findings suggested the use of *HOX* genes as a predictive marker for cancer metastasis.

### *HOX* gene as a biomarker for predicting disease recurrence

Despite significant advancements in oncopathology practice, the recurrence rate of many cancer types is high. Studies establishing the role of *HOX* genes as predictive biomarkers for cancer relapse are emerging. Among the notable ones, hypermethylated *HOXA9* and its downregulation is an independent prognostic marker as it was found to be significantly associated with the disease recurrence in NSCLC (Hwang et al. 2015). Contrary to this, *HOXA9* was shown to be overexpressed in patients of childhood leukemia, which showed a significant correlation with relapse (Adamaki et al. 2015).

High-grade serous ovarian cancer (HGSOC) patients who had a relapse following primary cytoreductive surgery and first-line adjuvant chemotherapy had *HOXA4/HOXB3* overexpression (Miller et al. 2018). Higher expression of *HOXB7* mRNA had been noted in patients diagnosed with HCC. HOXB7 promotes neoplastic progression via the bFGF-induced activation of the MAPK/ERK pathway implying a poor prognosis. The expression of *HOXB7* may be a promising candidate for recurrence prediction in HCC (Wang et al. 2017). In GC, *HOXC10* influences the malignant phenotype of GC cell lines, and its overexpression is correlated with hepatic and peritoneal recurrence (Miwa et al. 2019). The *HOX* gene expression profiling may prove to be a predictive biomarker of recurrence in various cancer subtypes.

### *HOX* genes in therapeutic resistance

Differential expression of *HOX* genes in various cell types and their ability to regulate a multitude of cellular functions in cancer has been extensively studied. The relapse and recurrence secondary to therapeutic resistance may be attributed to both intrinsic (transcriptomic, proteomic, epigenetic, and genetic) and extrinsic factors (pH, hypoxia, paracrine signaling, and tumor–host interactions) (Mansoori et al. 2017). Several *HOX* genes have been shown to render therapeutic resistance in various cancer subtypes. The downregulation of HOXA13 enhances the effectiveness of cisplatin chemotherapy in ESCC by inhibiting the expression of EMT markers such as *SNAI1* (Shi et al. 2018). However, HOXB7 has been shown to mediate cisplatin resistance in ESCC. Disrupting the HOXB7-PBX (pre-B cell leukemia transcription factor) complex by an inhibitory synthetic peptide HXR9 enhanced chemosensitivity to cisplatin proving *HOXB7* to be an effective target in ESCC (Zhou et al. 2020). Likewise, HOXB4 has been shown to render multidrug resistance of human myelogenous leukemia (HML) K562/ADM cells through the prolonged activation of the PI3K/AKT pathway. Knockdown of *HOXB4* could sensitize the HML cell lines by downregulating the expression of P-gp (P-glycoprotein), MRP (multidrug resistance-associated protein), and BCRP (breast cancer resistance protein) (Wang et al. 2016). Furthermore, HOXB7 and HOXB13 render BC cells resistant to tamoxifen through the stimulation of the EGFR and mTOR pathways, respectively (Jin et al. 2012; Shah et al. 2013). Another study on BC delineated the fact that overexpressed HOXD3 plays a crucial role in drug resistance and stemness via integrin β3-mediated Wnt/β-catenin signaling (Zhang et al. 2018b). Contarelli et al. (2020) reviewed that, among the genes in the HOXB cluster, HOXB9 had the capability in providing resistance to anti-angiogenic therapies in different cancer phenotypes (Contarelli et al. 2020). Moon et al. (2012) highlighted the role of HOXC6 in inducing the resistance to paclitaxel in HNSCC. The study demonstrated that HOXC6 modulates the expression of the anti-apoptotic gene *BCL2* to inhibit the normal apoptotic pathway (Moon et al. 2012). In NSCLC, HOXC8 has been considered as the promising therapeutic target to sensitize the cancer cells to cisplatin. HOXC8 functions as a transcriptional activator of the *TGFβ1* gene, and overexpression exaggerates normal TGF-β signaling, which could eventually promote the proliferation, migration, and growth of neoplastic cells (Liu et al. 2018). Sun et.al (2018) compared the expression levels of transcription factors that were associated with metastasis and cisplatin resistance in epithelial ovarian cancer. *HOXD8* was found to be upregulated in SKOV3-DDP cisplatin-resistant cells when compared with SKOV3 cisplatin-sensitive cells. They validated this observation in clinical samples which showed the overexpression of *HOXD8* in cisplatin resistance tissues when compared to primary malignant tumors, implying its potential role in cisplatin resistance (Sun et al. 2018b). Targeting the *HOX* genes or manipulating the expression of deregulated *HOX* genes is an imperative strategy for chemo-sensitization. However, *HOX* gene-mediated therapeutic resistance in different cancer phenotypes is poorly understood, warranting further studies.

## Conclusion and future perspectives

It has been well documented that *HOX* transcription factors play a crucial role in organogenesis and tissue homeostasis. They regulate a large number of targets that are particularly involved in key signaling pathways. In this review, we have highlighted the impact of deregulated *HOX* genes in cancer development and metastatic progression. Numerous molecular and clinical studies have demonstrated that any deregulation in the genes of HOX cluster would bring an imbalance in cellular pathways, which would eventually favor the cancer cells towards their metastatic progression. The integration of multiple signaling pathways regulated by *HOX* genes in cancer reflects their versatility in regulating diverse targets. Current literature supports that the genetic profiling of *HOX* gene clusters could be utilized as a clinical biomarker in early cancer detection. Hence, molecular techniques to determine the cancer stages are advantageous over the conventional method of cancer detection. Both in vitro and in vivo studies have proved that understanding the molecular mechanisms regulated by *HOX* genes and modulating aberrantly expressed *HOX* genes that provide resistance to therapies could be the most relevant strategy to sensitize the cancer cells for a better clinical outcome. Therefore, it would be an excellent opportunity for the clinical field to establish the targeted gene therapies against oncogenic molecules in cancer.

Even though the carcinogenic mechanism regulated by *HOX* genes is being extensively studied, studies are required to systematically understand the multifunctional role of *HOX* genes in each of the cancer types. Hence, we emphasize that the following research gaps could be filled to translate the findings into the clinical field.

- The genetic and epigenetic alterations in the HOX cluster genes and their mode of regulation are not studied in a variety of cancer types. Since those key alterations are responsible for driving metastasis, research should be focused on the molecular characterization of *HOX* genes at their transcriptional level.
- In addition to the solid tumors, the determination of *HOX* gene expression in body fluids such as lymph, serum, and saliva would allow clinicians for early cancer detection.
- To target a particular oncogenic *HOX* gene in cancer, more comprehensive studies are needed to understand the crosstalk between multiple signaling pathways regulated by *HOX* genes in cancer at both transcriptomic and proteome levels before their clinical translation.
- More than 75% of the HOX cluster-embedded ncRNAs are not yet functionally characterized. Since the functional interaction between ncRNAs and *HOX* genes is reported in some of the cancer types, research should focus on the characterization of the entire HOX cluster network.

## Data Availability

Not applicable.

## Abbreviations

ALL: Acute lymphoblastic leukemia
AML: Acute myeloid leukemia
BC: Breast cancer
CAFs: Cancer-associated fibroblasts
CC: Cervical cancer
CCC: Cholangiocellular carcinoma
CGH: Comparative genomic hybridization
CRC: Colorectal cancer
CSCC: Cutaneous squamous cell carcinoma
DFS: Disease-free survival
ECM: Extracellular matrix
EMT: Epithelial–mesenchymal transition
ESCC: Esophageal squamous cell carcinoma
GBM: Glioblastoma
GC: Gastric cancer
HCC: Hepatocellular carcinoma
HGSOC: High-grade serous ovarian cancer
HNSCC: Head and neck squamous cell carcinoma
HOX: Homeobox
IHC: Immunohistochemistry
LAC: Lung adenocarcinoma
lncRNA: Long non-coding RNA
MET: Mesenchymal–epithelial transition
miRNA: MicroRNA
MLL1: Mixed-lineage leukemia 1
ncRNAs: Non-coding RNAs
NSCLC: Non-small cell lung cancer
OS: Overall survival
OSCC: Oral squamous cell carcinoma
OVC: Ovarian cancer
PC: Pancreatic cancer
PCa: Prostate cancer
RBM: Retinoblastoma
T-ALL: T cell acute lymphoblastic leukemia
TNM: Tumor-node-metastasis
WGD: Whole-genome duplication
